# Long-term inhibition of ODC1 in APP/PS1 mice rescues amyloid pathology and switches astrocytes from a reactive to active state

**DOI:** 10.1186/s13041-024-01076-8

**Published:** 2024-01-12

**Authors:** Mridula Bhalla, C. Justin Lee

**Affiliations:** 1 Center for Cognition and Sociality, Life Science Institute (LSI), Institute for Basic Science (IBS), 55, Expo-ro, Yuseong-gu, Daejeon, 34126 Republic of Korea; 2 IBS School, University of Science and Technology (UST), 217 Gajeong-ro, Yuseong-gu, Daejeon, 34113 Republic of Korea

**Keywords:** Alzheimer’s disease, Reactive astrocytes, Ornithine decarboxylase 1

## Abstract

Alzheimer’s disease (AD) is characterized by the loss of memory due to aggregation of misphosphorylated tau and amyloid beta (Aβ) plaques in the brain, elevated release of inhibitory neurotransmitter gamma-aminobutyric acid (GABA) and reactive oxygen species from astrocytes, and subsequent neurodegeneration. Recently, it was found that enzyme Ornithine Decarboxylase 1 (ODC1) acts as a bridge between the astrocytic urea cycle and the putrescine-to-GABA conversion pathway in the brain of AD mouse models as well as human patients. In this study, we show that the long-term knockdown of astrocytic *Odc1* in APP/PS1 animals was sufficient to completely clear Aβ plaques in the hippocampus while simultaneously switching the astrocytes from a detrimental reactive state to a regenerative active state, characterized by proBDNF expression. Our experiments also reveal an effect of astrocytic ODC1 inhibition on the expression of genes involved in synapse pruning and organization, histone modification, apoptotic signaling and protein processing. These genes are previously known to be associated with astrocytic activation and together create a neuroregeneration-supportive environment in the brain. By inhibiting ODC1 for a long period of 3 months in AD mice, we demonstrate that the beneficial amyloid-clearing process of astrocytes can be completely segregated from the systemically harmful astrocytic response to insult. Our study reports an almost complete clearance of Aβ plaques by controlling an endogenous degradation process, which also modifies the astrocytic state to create a regeneration-supportive environment in the brain. These findings present the potential of modulating astrocytic clearance of Aβ as a powerful therapeutic strategy against AD.

## Introduction

**Fig. 1:**
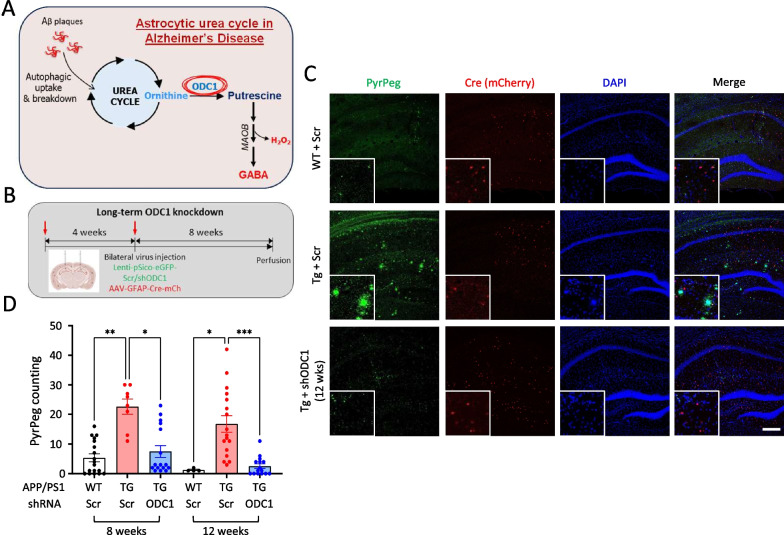
Long-term astrocytic KD of ODC1 almost completely clears Aβ plaques in APP/PS1 brains. **A** Schematic of the astrocytic urea cycle in Alzheimer’s Disease condition (adapted from Ju et al. [4]). **B** Schematic of the experimental timeline. Red arrows represent time points of virus injection. **C** Representative images of PyrPeg staining in the hippocampus of virus-injected APP/PS1 animals (Scale bar 200 μm). **D** Bar graph representing the number of plaques in the hippocampus of virus-injected APP/PS1 animals (each dot represents one brain slice, mouse N = 4–6 in each group). Data represents Mean ± SEM. *p < 0.05, **p < 0.01,***p < 0.001 (Kruskal–Wallis test and Dunn’s multiple comparisons test)

Ornithine decarboxylase 1 (ODC1) is well known for its function in the synthesis of putrescine from L-ornithine in hepatocytes [1], the rate-limiting step in the synthesis of polyamines spermine and spermidine [2]. ODC1 dysregulation has been associated with liver cancer and prostate cancer, and several studies have reported changes in the transcription, translation and degradation of ODC1 during carcinogenesis [3]. Recently, it was reported that in astroglia of the brain, ODC1 was involved in the conversion of ornithine to putrescine, which further produced GABA and lead to memory impairment in Alzheimer’s disease (AD) [4]. ODC1 showed disease-specific upregulation in the hippocampal astrocytes of APP/PS1 mice and human AD patients, and genetic knockdown (KD) of the enzyme was able to reduce hypertrophy, putrescine and GABA accumulation in the astrocytes while also improving memory and synaptic firing. This report also suggested that genetic KD of ODC1 in astrocytes was able to successfully clear amyloid beta (Aβ) accumulation without producing excessive inhibitory neurotransmitter GABA upto 4 weeks after genetic KD. With the knowledge that ODC1 acts as a switch between beneficial astrocytic function of Aβ clearance and the detrimental GABA-producing pathway (Fig. 1A), we sought to uncover if long-term KD of this enzyme in the astrocytes could potentially switch astrocytes from a neurodegenerative reactive state to a neuroprotective state.

## Materials and methods

### Animal husbandry

All animal experiments were performed on a mouse model for AD carrying mutations APPswe/PSEN1dE9 (APP/PS1 mice of B6C3 hybrid background) to mimic amyloidosis (RRID: MMRRC_034829-JAX) originated from Jackson Laboratory (USA, stock number 004462). Mouse genotype was verified by performing PCR using the following primers: APP/PS1_F-5′ AAT AGA GAA CGG CAG GAG CA 3′; APP/PS1_R-5′ GCC ATG AGG GCA CTA ATC AT 3′. 10–12 month old animals of both sexes were used for the virus injection experiment, transgenic mice matched with WT littermates. Animals were maintained in a 12:12 light dark cycle (lights out at 20:00) and provided with food pellets and water ad libitum*.* Care and handling of animals were in accordance with the guidelines of the Institutional Animal Care and Use Committee of IBS (Daejeon, South Korea).

### Virus injection

Animals were anesthetized with isoflurane and placed on stereotaxic frames (Kopf). The scalp was incised, and a hole was drilled into the skull above the dentate gyrus (A/P, − 1.5 mm; M/L, − 1.2 or + 1.2 mm from bregma, D/V, − 1.85 mm from the skull surface). The virus was loaded into a stainless-steel blunt needle (World Precision Instruments) and injected bilaterally into the dentate gyrus at a rate of 0.1 μl min− 1 for 10 min (total 1 μl in each hemisphere) using a syringe pump (KD Scientific). Viruses were generated at the Institute for Basic Science virus facility (IBS Virus Facility). AAV-GFAP-Cre-mCh, Lenti-pSico-GFP-Scr, Lenti-pSico-GFP-shODC1 viruses were used in each experiment (shRNA sequences from [4]). Mice were perfused and used for immunohistochemistry 8- and 12-weeks after injection.

### Immunohistochemistry

Mice were anaesthetized with isoflurane and perfused with 0.9% saline followed by ice-cold 4% paraformaldehyde (PFA). Excised brains were post-fixed overnight at 4 °C in 4% PFA and dehydrolyzed in 30% sucrose for 48 h. Coronal hippocampal sections of 30 μm thickness were prepared in a cryostat and stored at 4 °C. Before staining, sections were thoroughly washed in 0.1 M PBS and incubated for 1 h in blocking solution (0.3% Triton X-100, 4% Donkey Serum in 0.1 M PBS). Primary antibodies were added to blocking solution at desired dilution and slices were incubated in a rocker at 4 °C overnight. Unbound antibodies were washed off using 0.1 M PBS (3 times), followed by corresponding secondary antibody incubation (in blocking solution) for 1 h at room temperature. PyrPeg was prepared and incubated as a secondary antibody (RT for 1 h). Unbound antibodies were washed with 0.1 M PBS (3 times) and DAPI was added to PBS (1:1000 dilution) in the second step to visualize the nuclei of the cells. Sections were mounted with fluorescent mounting medium (Dako) and dried. Series of fluorescent images were obtained using the Zeiss LSM900 microscope.

22-μm Z stack images in 2 μm steps were processed for Sholl analysis using the ZEN Digital Imaging for Light Microscopy blue system (Zeiss, ver. 3.2) and ImageJ (NIH, ver. 1.54b.) software. Primary antibodies were diluted to the following amounts: chicken anti-GFAP (Millipore, AB5541) 1:500; rabbit anti-proBDNF (Alomone lab, ANT-006) 1:200; and PyrPeg (0.1 μM). Secondary antibodies (Jackson) were diluted 1:500 in the blocking solution.

### Sholl analysis

Sholl analysis was performed on serially stacked and maximally projected confocal images as previously described [5]. Z-stacked images (22 μm stack, 2 μm steps) of brain sections immunostained with GFAP antibody were used for Sholl analysis. The Sholl analysis plugin applied in ImageJ (NIH) constructs serially concentric circles at 5 μm intervals from the center of GFAP signal (soma) to the end of the most distal process of each astrocyte. The number of intercepts of GFAP-positive processes at each circle and the radius of the largest circle intercepting the astrocyte were analyzed and reported.

### NGS data analysis

FastQ files obtained from the NCBI GEO Accession ID GSE185916 were imported to Partek Genomics Suite (Flow ver. 10.0.21.0328; Copyright 2009, Partek, St. Louis, MO, USA), where further processing was carried out. Read quality was confirmed for each sample using FastQC. High-quality reads were aligned using STAR (2.7.3a). Aligned reads were quantified to the mouse genome assembly (mm10, RefSeq transcripts assembly 93) and normalized to the median ratio (for analysis using DeSeq2). Differential analysis was carried out using DeSeq2. Gene ontology analysis was carried out from Median Ratio-normalized counts with reference to the default KEGG geneset database.

### Statistical summary

All analyses were done blindly. The number of experimental samples for each group are listed in the figure legends. Numbers and individual dots refer to individual samples (individual cells) unless clarified otherwise in figure legends. N represents number of animals used for the experiment. Data representation and statistical analysis was performed using GraphPad Prism (Graphpad Software). For image analysis, ImageJ (NIH) was used**.** Statistical significance was set at ∗ p < 0.5, ∗  ∗ p > 0.01, ∗  ∗  ∗ p < 0.001 and ∗  ∗  ∗  ∗ p < 0.0001 (unless mentioned otherwise in figure legends).

## Results

To test our hypothesis, we injected viruses carrying Cre-driven Scrambled construct or shRNA targeting *Odc1* (Lenti-pSico-GFP-shODC1/Scr) along with GFAP-promoter-driven Cre recombinase (AAV-GFAP-Cre-mCh) to induce astrocyte-specific knockdown (KD) of ODC1 in the hippocampus of 10–12-month-old APP/PS1 animals. We injected viruses in 2 sets of animals, at different time points (4 weeks apart) and performed immunohistochemistry at 8- and 12-weeks post-injection respectively (Fig. 1B). Upon immunostaining with PyrPeg, a fluorescent molecule which can selectively detect Aβ plaques [6], we were able to observe a 60% reduction in plaque numbers at 8-week post-injection. Surprisingly, we observed almost complete clearance of accumulated Aβ aggregations in the hippocampus of APP/PS1 animals 12 weeks after KD (Fig. 1C, D). This is a significant improvement in Aβ clearance compared to the 40% reduction 4 weeks after KD, as previously reported [4].

**Fig. 2:**
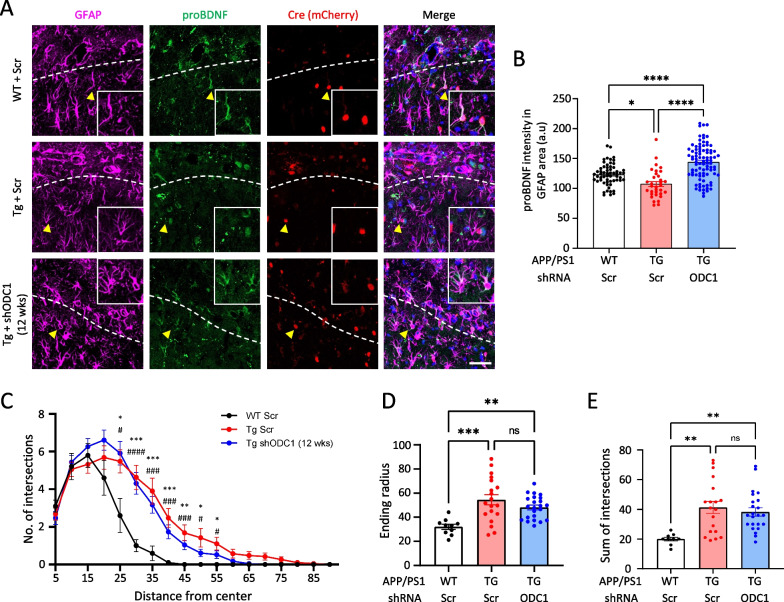
Long-term ODC1 KD switches astrocytes from reactive to active state in APP/PS1 mice. **A** Representative images of proBDNF and GFAP-immunoreactive cells in the hippocampus of virus-injected APP/PS1 animals (Scale bar 40 μm, white dashed line represents the interface between CA1 *layer lacunosum moleculare* and molecular layer of DG). **B** Bar graph representing the intensity of proBDNF in the GFAP-positive cells from images in panel A (Ordinary one-way ANOVA with Sidak’s multiple comparisons test). **C** Average number of intersections of the astrocytic branches with concentric circles in Sholl analysis from images in panel A (RM two-way ANOVA with Geisser-Greenhouse correction with Tukey’s multiple comparisons test; *p value for WTScr vs TgScr; #p value for WTScr vs TgODC1; no significant differences between TgScr and TgODC1). **D** Bar graph representing the ending radius of concentric circles around astrocyte branches in Sholl analysis (Ordinary one-way ANOVA with Tukey’s multiple comparisons test). **E** Bar graph representing the sum of intersections made by the astrocyte branches and concentric circles in Sholl analysis (Ordinary one-way ANOVA with Tukey’s multiple comparisons test). Data represents Mean ± SEM. # or*p < 0.05, ## or **p < 0.01,### or ***p < 0.001, #### or ****p < 0.0001

Previous studies have reported the transformation of resting astrocytes to active state in the presence of non-aversive and environmentally-beneficial conditions [7]. These active astrocytes can be characterized by the expression of proBDNF, a growth factor which can be linked to enhanced cognition and neuroplasticity [8] as well as increased astrocytic hypertrophy, likely due to increased metabolism within the cells. We examined the expression of proBDNF in the astrocytes of APP/PS1 animals and compared them against the long-term ODC1 KD condition (Fig. 2A, B). We observed that 12 weeks after ODC1 KD, the astrocytes in the hippocampal CA1 *layer lacunosum moleculare* (above white dashed line, Fig. 2A) and molecular layer of the dentate gyrus (below white dashed line, Fig. 2A) of APP/PS1 animals showed increased levels of proBDNF as compared to the Scr-injected group (Fig. 2B). This increase was particularly seen in the astrocytes expressing the mCherry-tagged Cre protein (yellow arrowheads, Fig. 2A), indicating that this was indeed an effect of ODC1 genetic KD within the cell. Furthermore, Sholl analysis of the astrocytes in these hippocampi revealed that there was no significant difference in astrocytic hypertrophy, sum of intersections and ending radius in Scr-injected and shODC1-injected APP/PS1 TG mice (Fig. 2C–E). This is reminiscent of the active astrocytes found in enriched environment (EE)- housed animals [7], known to contribute to increased synaptogenesis and neuroplasticity via proBDNF release [9–11].

**Fig. 3:**
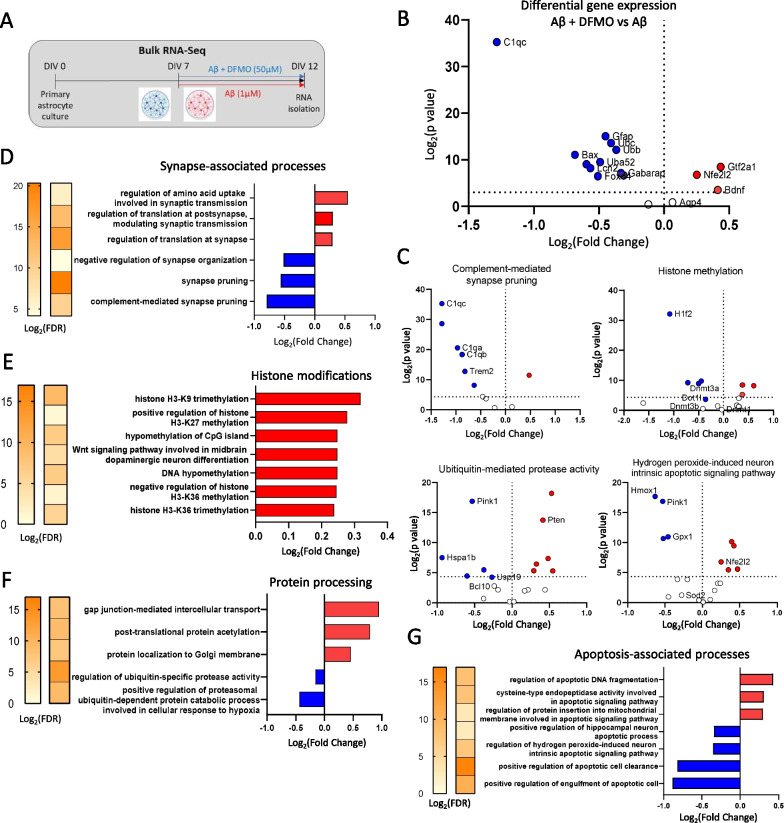
Transcriptome of long-term ODC1-inhibited astrocytes shows dramatic changes, resembling enriched environment-house animal brains. **A** Experimental timeline schematic for bulk RNA sequencing of primary astrocyte culture. **B** Volcano plot representing the differential expression of specific genes associated with astrocyte reactivity, learning and memory, apoptosis, transcriptional regulation, ubiquitination and autophagy from bulk RNA-Seq. Horizontal dotted line refers to p < 0.05, vertical dotted line indicates no change in expression level). **C** Volcano plots representing the differential expression of genes involved in different GO terms from the bulk-RNASeq dataset. Horizontal dotted line refers to p < 0.05, vertical dotted line indicates no change in expression level. **D** Gene ontology analysis for synapse-associated genes from bulk RNA-Seq of Aβ and Aβ + DFMO-treated primary cultured astrocytes. **E** Gene ontology analysis for histone modification-associated genes from bulk RNA-Seq of Aβ and Aβ + DFMO-treated primary cultured astrocytes. **F** Gene ontology analysis for protein processing pathways and related genes from bulk RNA-Seq of Aβ and Aβ + DFMO-treated primary cultured astrocytes. **G** Gene ontology analysis for genes associated with apoptosis from bulk RNA-Seq of Aβ and Aβ + DFMO-treated primary cultured astrocytes

To study the changes in astrocytic transcriptome after long-term ODC1 inhibition, we analyzed Next Generation Sequencing (NGS) data from RNA isolated from primary astrocyte cultures treated with oligomerized Aβ (1 μM) in the presence or absence of ODC1 inhibitor diflouromethylornithine (DFMO, 50 μM) for 5 days (Fig. 3A) [4]. Previous studies have reported that *Nrf2* activation can upregulate BDNF in vitro [12]. Differential gene expression analysis from our NGS data revealed the upregulation of Brain Derived Neurotrophic Factor (*Bdnf*) as well as transcription regulatory genes *Nrf2 (Nfe2l2)* and *Gtf2a1* in DFMO-treated AD-like astrocytes (Fig. 3B). While not statistically significant, *Aqp4* was also upregulated in DFMO-treated condition, indicating an improved learning and memory-supportive astrocytic state [8]. Astrocyte reactivity markers, such as *Gfap* and *Lcn2* were downregulated, as were apoptosis and autophagy-associated genes *Gabarap, Bax* and *Foxo4* (Fig. 3B). *C1qc* was also found to be downregulated, suggesting that complement-associated synaptic pruning was reduced [13]. This was supported by the gene ontology (GO) data which indicated a downregulation of synapse-pruning processes along with upregulation of synaptic transmission-related processes (Fig. 3D). Previous reports have also suggested an increase in neuronal growth and synapse-associated genes and decreased apoptotic signaling and proteolysis in the brains of mice housed in enriched environments [14]. Interestingly, histone modification-associated processes were greatly upregulated, indicating a massive transcriptional change in AD-like astrocytes after long-term ODC1 inhibition (Fig. 3E). GO analysis of the RNASeq data reveals that changes caused by DFMO treatment of AD-like astrocytes resemble the genetic changes from EE housing in the mouse brain [14] (Fig. 3D, E, F and G). Apoptotic pathways affecting neurons were downregulated as were proteolysis-associated processes (Fig. 3F, G), while post-translational protein modifications were upregulated (Fig. 3F). Differential gene expression analysis revealed significant downregulation of several genes involved in the GO terms “complement-mediated synapse pruning”, “ubiquitin-mediated protease activity”, “histone methylation” and “hydrogen peroxide-induced neuron intrinsic apoptotic signaling pathway” (Fig. 3C). Taken together, the differential gene expression and GO analysis revealed that the transcriptome of long-term ODC1-inhibited AD-like astrocytes resembles active astrocytes from EE-like conditions.

## Discussion

**Fig. 4:**
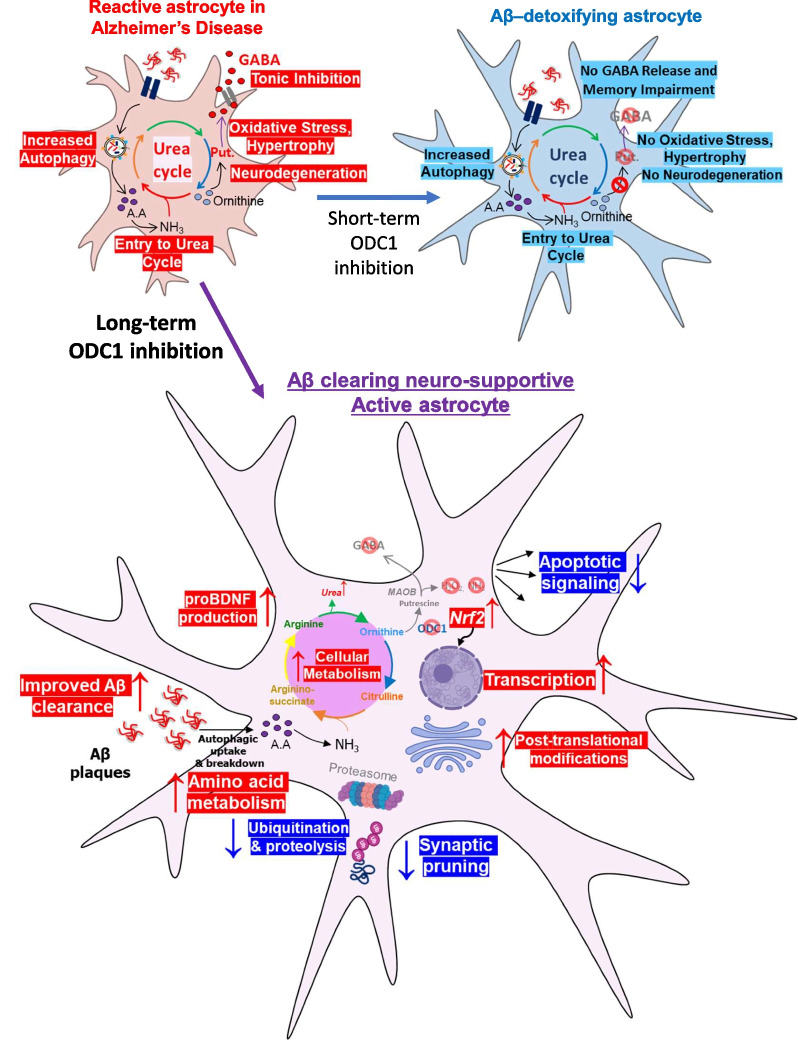
Long-term inhibition of ODC1 in AD creates an Aβ-clearing and neuro-supportive active state. Top-Left: In AD, astrocytes show upregulated autophagy to uptake accumulated amyloid deposits and switch-on the urea cycle along with upregulation of enzyme ODC1, which increases putrescine, GABA, H_2_O_2_ and ammonia production, causing neurodegeneration and memory impairment. Top-Right: Short-term ODC1 inhibition in AD mouse models blocks the conversion of ornithine to putrescine, increases the flux of the urea cycle, reduces production of putrescine, GABA, H_2_O_2_ and thereby preventing oxidative stress, neurodegeneration, and memory impairment. These astrocytes can be called Aβ-detoxifying astrocytes (schematics adapted from Ju et al*.* [4]) Bottom: Long-term inhibition of ODC1 in AD-like astrocytes can improve Aβ plaque clearance and drastically alter the transcriptome of astrocytes, by increasing histone modifications, *Nrf2* expression, proBDNF production, protein-associated processes and cellular metabolism. These changes, along with reduced apoptotic signaling, ubiquitination and synaptic pruning-associated genes, resemble the transcriptome of EE active astrocytes and can create a neuro-supportive environment for regeneration (Created using biorender.com)

This study highlights the dichotomous role of ODC1 as the switch between beneficial and detrimental astrocytic processes in Alzheimer’s disease. We have previously shown that ODC1 inhibition rescues memory deficits and reduces aberrant astrocytic GABA production in APP/PS1 animals [4]. In this study, we find that long-term genetic inhibition of astrocytic ODC1 in the hippocampus can completely remove the Aβ plaque load from the brains of APP/PS1 mice (Fig. 1). We unveil that long-term inhibition of ODC1 can switch astrocytes from a disease-causing neurodegenerative reactive state to a beneficial and neuro-supportive active state, by expressing BDNF precursor protein proBDNF and displaying a hypertrophic morphology (Fig. 2). In-vitro NGS and GO analysis reveals massive changes in the transcriptome of long-term ODC1-inhibited astrocytes, which can be seen as changes in histone modifications and key protein processing genes, as well as increased synapse-supportive processes and reduced apoptotic signaling (Fig. 3). Taking our current data together with previous reports, we state that the long-term inhibition of ODC1 can have a significantly positive effect on AD pathology, not just by facilitating complete Aβ plaque clearance without GABA and H_2_O_2_ production, but also by initiating synapse supportive and anti-apoptotic processes within the astrocytes, thereby supporting neuroregeneration (Fig. 4).

Previous studies have reported morphological hypertrophy of hippocampal astrocytes [15], as well as proBDNF expression in astrocytes [7] in the mouse brain after EE housing. These astrocytes have been referred to as “active astrocytes” and create a neuro-regeneration supportive environment in the brain, attributed to the altered brain volume and mitochondrial activity associated with EE [8, 16]. In contrast, reactive astrocytes have always been found associated with pathologies, such as Alzheimer’s disease, traumatic brain injuries, stroke, and are known to have reduced proBDNF expression and release GABA and ROS, contributing to neurodegeneration [17]. Several studies report that frequent and regular exercise can exacerbate astrogliosis and degeneration in the hippocampus of AD transgenic animals [11, 18]. Our data shows that long-term genetic inhibition of ODC1 in reactive astrocytes of APP/PS1 animals induces the expression of proBDNF and hypertrophic morphology of astrocytes (Fig. 2), similar to the effects of EE housing [7]. Comparing our NGS data against DNA Chip study performed from whole brain lysates of EE housed mice [14], we can infer a congruence in the transcriptomic changes of long-term ODC1-inhibited astrocytes and EE brains. It has been reported that EE housing can ameliorate memory deficits in APPSwe animals but also causes an increase Aβ plaque accumulation [19]. In contrast, while long-term ODC1 KD astrocytes resemble a hypertrophied active state similar to EE astrocytes in our study (Fig. 2), we do not see any increase in Aβ accumulation in the APP/PS1 mouse brains (Fig. 1). This could be due to the continuous and efficient astrocytic clearance by the upregulation of urea cycle after ODC1 inhibition, as reported by Ju et al.[4]. In addition to proBDNF production, our data also reveals a plethora of transcriptomic changes in vital processes of the cell, such as histone modification, protein post-translation modification and ubiquitination (Fig. 3). These changes, along with the marked downregulation of synaptic pruning processes and upregulation of synapse-supporting processes, together indicate a healthy and supportive environment for neuroregeneration, created by the long-term inhibition of ODC1 in AD-like astrocytes. Together with our proBDNF immunostaining data, we propose that long term ODC1 inhibition in AD-like astrocytes can switch them to a regeneration-supportive state, potentially having beneficial effects similar to those previously reported to be associated with EE housing [10].

Adding to the previously reported positive role of ODC1 inhibition, we conclude that long-term astrocytic inhibition of ODC1 in AD not only halts neurodegeneration by reducing the production of toxic by-products from the urea cycle [4] but also supports regeneration by reversing the metabolic state of astrocytes from a toxic reactive state to a beneficial, neuro-supportive state. These promising new findings and their potential to regenerate neurons in light of AD-associated neurodegeneration remains to be explored. Armed with this knowledge, we propose long-term and continuous inhibition of astrocytic ODC1 as a powerful therapeutic tool to reverse AD-associated neurodegeneration.

## Data Availability

The NGS dataset supporting the conclusions of this article is available in the NCBI GEO repository, https://www.ncbi.nlm.nih.gov/geo; GEO: GSE185916. The viruses used in this study were provided by and will be available with the Institute for Basic Science Virus Facility (https://www.ibs.re.kr/virusfacility/, Daejeon, South Korea) upon request. Further information and requests for raw data files and resources should be directed to and will be fulfilled by the lead contact Dr C Justin Lee (cjl@ibs.re.kr).

## Abbreviations

Aβ: Amyloid beta
AD: Alzheimer’s disease
BDNF: Brain derived neurotrophic factor
DFMO: Difluoromethylornithine
EE: Enriched environment
GABA: Gamma-aminobutyric acid
GO: Gene ontology
KD: Knockdown
NGS: Next generation sequencing
*Nrf2*: Nuclear factor erythroid-derived 2-related factor 2 (NFE2L2)
ODC1: Ornithine decarboxylase 1
RNASeq: RNA sequencing
